# Stronger vection in junior high school children than in adults

**DOI:** 10.3389/fpsyg.2014.00563

**Published:** 2014-06-12

**Authors:** Nobu Shirai, Tomoko Imura, Rio Tamura, Takeharu Seno

**Affiliations:** ^1^Department of Psychology, Faculty of Humanities, Niigata UniversityNiigata, Japan; ^2^Department of Information Systems, Niigata University of International and Information StudiesNiigata, Japan; ^3^Faculty of Design, Kyushu UniversityFukuoka, Japan; ^4^Institute for Advanced Study, Kyushu UniversityFukuoka, Japan; ^5^Research Center for Applied Perceptual Science, Kyushu UniversityFukuoka, Japan

**Keywords:** vection, development, junior high school-aged children, adults, self-motion

## Abstract

Previous studies have shown that even elementary school-aged children (7 and 11 years old) experience visually induced perception of illusory self-motion (vection) (Lepecq et al., 1995, *Perception*, 24, 435–449) and that children of a similar age (mean age = 9.2 years) experience more rapid and stronger vection than do adults (Shirai et al., 2012, *Perception*, 41, 1399–1402). These findings imply that although elementary school-aged children experience vection, this ability is subject to further development. To examine the subsequent development of vection, we compared junior high school students' (*N* = 11, mean age = 14.4 years) and adults' (*N* = 10, mean age = 22.2 years) experiences of vection. Junior high school students reported significantly stronger vection than did adults, suggesting that the perceptual experience of junior high school students differs from that of adults with regard to vection and that this ability undergoes gradual changes over a relatively long period of development.

## Introduction

Exposure to a moving visual field that simulates the retinal optical flow generated by self-movement usually causes the perception that one's own body is moving. This phenomenon is known as “vection” (Fischer and Kornmuüller, 1930). For example, when a stationary person observes a train beginning to move, s/he is likely to perceive that s/he is moving in the direction opposite to that of the train. This phenomenon, known as the “train illusion,” is a good example of vection (e.g., Seno and Fukuda, 2012).

The first scientific experiment examining vection was conducted in 1973 by Brandt et al. (1973). Since that time, the stimulus characteristics necessary for the induction of vection have been investigated extensively (e.g., Seno et al., 2009; Riecke, 2010; Palmisano et al., 2011). For instance, the effect of the size of visual stimuli on vection induction has been a major topic of vection research since the initial study conducted by Brandt et al. (1973). It has been consistently reported that a visual stimulus encompassing a wider visual field induces stronger vection (e.g., Brandt et al., 1973; Held et al., 1975; Lestienne et al., 1977) and that the peripheral visual field is more effective for vection induction than is the central visual field (Brandt et al., 1973; Held et al., 1975; Johansson, 1977; Dichgans and Brandt, 1978).

The temporal aspects of vection have been also extensively investigated. Vection induction requires a “latency” period (Kennedy et al., 1995) that usually lasts about 4–12 s (e.g., Bubka et al., 2008). When subjectively stronger vection is obtained, the latency tends to be shorter. Another important temporal aspect of vection is its “duration.” Vection disappears and reappears during periods of stimulus presentation; the cumulative period of vection, referred to as the “duration” of vection, is also an important measure of this phenomenon. Stronger vection tends to be associated with longer duration. Although latency and duration may appear to be negatively correlated, they are completely different phenomena. Thus, it is important to measure both latency and duration.

Another well-known property of vection is the contribution of sensory inputs from multiple modalities. The multiple modalities are all related to self-motion perception (Gibson, 1966), and their inputs are generally harmoniously integrated (Rieser et al., 1995). Thus, vection is generally facilitated by consistent information about potential self-motion from other modalities. For instance, consistent vestibular input (Wright, 2009), head movements (Ash et al., 2011), and locomotion (Seno et al., 2011a,b) facilitate vection. Furthermore, consistent somatosensory cues applied to a hand also facilitate vection (Lécuyer et al., 2004), and air flow toward an observer's face facilitates forward vection (Seno et al., 2011b). Finally, vection is facilitated by subsonic vibrations that are consistent with visual rotation (Riecke et al., 2008, 2009). Indeed, many studies have demonstrated that multiple inputs consistent with visual optic flow can facilitate vection. In this paper, we conclude that vection can be mediated by multiple modalities.

The ability to process inputs from multiple modalities to detect and control self-movement seems to develop relatively early in life. The fact that significant postural compensation in response to large visual motion patterns develops at least between 7 and 9 months of age (e.g., Bertenthal and Bai, 1989; Bertenthal et al., 1997) suggests that even young infants can detect visual motion patterns and utilize these patterns to control their self-movement. The ability to achieve postural compensation becomes increasingly sophisticated through individual experiences of voluntary locomotion; infants who have experienced voluntary locomotion show more systematically compensational body movements than do infants who have not experienced voluntary locomotion (Higgins et al., 1996). This means that the development of the ability to detect self-movement from visual information is promoted or shaped by the experience of non-visual (e.g., vestibular and/or proprioceptive) inputs related to self-movements. In contrast, a recent study indicated that particular developmental changes in visual motion perception occur suddenly, immediately before the onset of voluntary locomotion in infancy (Shirai and Imura, 2014). Thus, visual development seems to lead and promote the development of the non-visual ability to control several aspects of self-movement. In summary, infant studies show that abilities related to processing visual and non-visual inputs pertinent to self-movement are developed by bidirectional interactions involving experiences of visual and non-visual inputs during infancy.

Whereas the interaction between visual and non-visual inputs in infancy have been thoroughly investigated, the later development of such interactions, including vection, has been relatively neglected. For instance, only a few empirical studies have addressed the development of vection. Lepecq et al. (1995), who conducted the first empirical study of vection in young children, reported that vection could occur in 7–11-year-old children. They also found that about half of the 7-year-old children, but most of the 11-year-old children, reported experiences of vection under the experimental condition. Their results suggest that experiences of vection can occur as young as elementary school and that the ability to perceive vection is still developing between early and late childhood. More recently, Shirai et al. (2012) used an experimental approach to examine the development of vection, directly comparing vection among elementary school students and adults under the same experimental conditions. They measured the latency, duration, and magnitude of the vection elicited by a large field with an expanding radial flow pattern in elementary school students (mean age = 9.2 years) and adults (mean age = 21.5 years). Their results indicated that the elementary school students reported vection experiences of shorter latency and stronger magnitude than those of adults.

These previous developmental studies carry two main implications about the development of vection: (1) valid vection can be observed in children as young elementary school age and (2) vection may continue to develop beyond childhood. However, certain aspects of the development of vection in human beings remain unclear, and these warrant additional research attention. For instance, many studies have shown that visual motion perception develops relatively slowly after childhood (e.g., Schrauf et al., 1999; Ellemberg et al., 2003; Hadad et al., 2011), whereas even children of elementary and junior high school age tend to rely more on visual than on vestibular and/or proprioceptive information to maintain their postural stability (e.g., Hirabayashi and Iwasaki, 1995; Rival et al., 2005). These findings lead the expectation that the characteristics of vection, the vision-based perception of self-movement, change dynamically beyond childhood. To expand our knowledge about the development of vection, we examined children older (junior high school students) than those who participated in the study conducted by Shirai et al. (2012) with the goal that our series of studies examining the relationship between development and vection can serve as a foundation for future vection research.

## Materials and methods

### Ethics statements

The present study was approved by the Ethics Committee for Psychological Research at Niigata University and was conducted following the Helsinki Declaration. Written informed consent was obtained from all participants (and their parents, in cases of children).

### Participants

Ten adult (undergraduate students, mean age = 22.22 years, range = 21.67–22.67 years) and 11 junior high school students (mean age = 14.39 years, range + 13.08–15.58 years) participated in the experiment. All participants were healthy and had normal or corrected-to-normal vision and no history of vestibular system disease. None of the participants was aware of the purpose of the experiment.

### Apparatus, stimuli, and experimental conditions

The experiment was conducted in a dark room, and visual stimuli were generated and controlled by a computer (MB543J/A, Apple). The stimuli were displayed on a 50-inch video screen (Vsync = 60 Hz, resolution = 1024 × 768 pixels) using a digital light-processing (DLP) projector (BenQ MX511). The stimuli projected on the screen were identical to those used by Seno et al. (2010) and Shirai et al. (2012); an expanding optical flow pattern was created by randomly positioning 16,000 dots inside a simulated cube and moving the observer's viewpoint to simulate forward self-motion at 16 m/s. We used two conditions, large field and small field, which varied according to stimulus size. Under the large-field condition, a visual stimulus was projected on the full area of the screen (102 × 76 °). Under the small-field condition, a visual stimulus appeared only in the central circular area subtending 40°. Thus, the size of the visual stimulus under the small-field condition was about 16% of that under the large-field condition. Previous studies (Brandt et al., 1973; Seno et al., 2013a,b) have shown that the magnitude of vection tends to be much weaker in response to a smaller visual stimulus. Thus, the experimental condition involving the smaller visual stimulus was used as the standard under the large-field condition to test whether the participants' perceptions of vection under the current experimental conditions were consistent with previous results regarding this phenomenon. If our results were inconsistent with previous findings, participants' perceptions of vection under the large- and small-field conditions would not significantly differ. In contrast, if participants' perceptions of vection were consistent with extant understandings, they would report greater vection under the large-field than under the small-field condition.

### Procedure

Five trials were conducted under each condition. Hence, each participant engaged in a total of 10 trials. In each trial, a visual stimulus was presented for 40 s. Participants sat on a chair in front of the screen at a viewing distance of about 57 cm. No head or chin rest was used, leaving the head and chin of participants unrestrained. Although no fixation point was presented, participants were instructed to fixate on the center of the radial flow pattern during each trial while remaining relaxed. Participants' task was to press a computer mouse button connected to the computer when they perceived self-motion. This allowed us to record the latency of vection onset and the cumulative duration of the observers' vection experience during each trial. At the end of each trial, the participants rated the magnitude of vection using a visual analog scale, a 10-cm line segment drawn on paper. The observers indicated their responses by drawing a short orthogonal line segment to estimate the magnitude of vection. The distance (in mm) from the left edge of the scale to the intersection was adopted as the index of vection magnitude; longer distance indicated stronger vection. Before the start of experimental sessions, the following instructions were given to all participants: “After each trial, please rate the magnitude of your experience of self-movement during the trial. When you think that your body movement was weakest, that is, you felt your body did not move forward at all, please draw a short vertical line at the left edge of the long line. When you think your body movement was strongest, that is, you felt as if your body really moved forward, please draw a short line at the right edge of the long line. In cases where you felt that the magnitude of your body movement was at a point somewhere between the weakest and strongest states, please draw a short line at the point on the long line that corresponds to your perception.” Although visual analog scales seem to be less popular than direct reports (e.g., verbal responses) as a means of measuring the magnitude of vection, we decided to adopt a visual analog scale for the following reasons. First, our previous informal observations identified several difficulties with the use of verbal reports by young children, as young children often provided inconsistent oral reports about the magnitude of vection. For instance, some children reported huge or negative numbers in a joking manner despite being instructed to estimate the magnitude using a scale from 0 to 100. The use of a visual analog scale is an effective way to avoid such inappropriate responses, and our previous study succeeded in measuring the magnitude of vection among young children by using such an approach (Shirai et al., 2012). Additionally, it has been shown that children develop the ability to use a visual analog scale to represent a rating by early elementary school age; children of about 7 years of age can understand the linear relationship between an arbitrary number and a position on a visual analog scale and can accurately estimate a given number using such a scale (Booth and Siegler, 2006). Thus, a visual analog scale is a valid tool for measuring the magnitude of vection, even among children.

### Results and discussion

**Figure 1:**
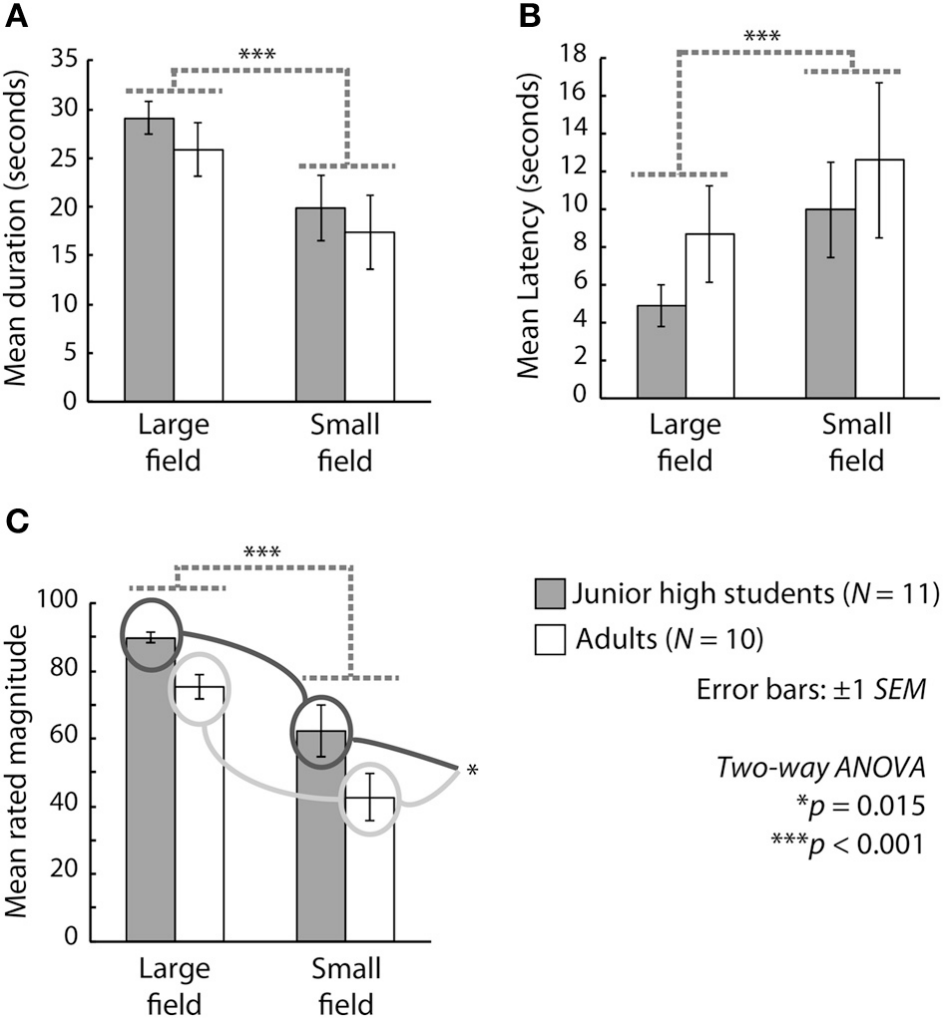
**Mean (A) duration, (B) latency, and (C) magnitude of vection across participants**. Gray and white bars show the results for junior high school students and adults, respectively. The two bars on the left side and those on the right side of each graph indicate results under the large-field and the small-field conditions, respectively. Error bars represent ±1 s.e.m.

Figure 1 presents the results in terms of duration, latency, and magnitude. We conducted a Two-Way ANOVA (age group vs. stimulus size) for each outcome measure. With respect to the mean duration of vection, the main effect of stimulus size was significant [*F*_(1, 19)_ = 34.281, *p* < 0.001, η^2^_*p*_ = 0.643], but the main effect of age and the interaction between age and stimulus size were not significant [*F*_(1, 19)_ = 0.531, *p* > 0.1, η^2^_*p*_ = 0.027; *F*_(1, 19)_ = 0.063, *p* < 0.001, η^2^_*p*_ = 0.003, respectively]. In terms of the mean latency of vection, the main effect of stimulus size was significant [*F*_(1, 19)_ = 8.488, *p* = 0.0089, η^2^_*p*_ = 0.308], but the main effect of age and the interaction between age and stimulus size were not significant [*F*_(1, 19)_ = 0.817, *p* > 0.1, η^2^_*p*_ = 0.041; *F*_(1, 19)_ = 0.140, *p* > 0.1, η^2^_*p*_ = 0.007. respectively]. With regard to the mean magnitude of vection, the main effects of stimulus size [*F*_(1, 19)_ = 42.712, *p* < 0.001, η^2^_*p*_ = 0.692] and age [*F*_(1, 19)_ = 7.143, *p* = 0.0150, η^2^_*p*_ = 0.273] were significant, but the interaction between age and stimulus size was not significant [*F*_(1, 19)_ = 0.312, *p* > 0.1, η^2^_*p*_ = 0.016].

The significant main effect of stimulus size and the lack of a significant interaction between age and stimulus size for all outcome measures indicate that both children and adults experienced more stable, rapid, and stronger vection under the large-field than under the small-field condition. Thus, the vection experiences of junior high school students appear to have been similar to those of adults under the present experimental conditions. On the other hand, the results for the mean magnitude of vection indicated significantly stronger experiences among the students than among the adults, with the junior high school students perceiving stronger vection than the adults did even though the mean latency and duration of vection were equivalent in these two groups. These results are partly consistent with those reported by Shirai et al. (2012), who investigated vection in elementary school students and adults. They reported that the latency and magnitude (but not the duration) of vection were shorter and larger, respectively, in elementary school students than in adults. The present results are consistent with those of Shirai et al. (2012) in terms of magnitude but inconsistent in terms of latency (for a comparison with the present results, see Figure 1 of Shirai et al., 2012, in particular). Thus, it is plausible that the experiences of vection in junior high school-aged children differ from those in both younger (e.g., elementary school-aged) and older (adult) individuals and that the age period associated with junior high school may be a developmental midpoint between elementary school and adulthood with regard to the development of vection.

## General discussion

According to the results of this study, although the tendency of junior high school students to perceive self-motion in response to visual stimuli is, in some respects, comparable to that of adults, the experiences of the two groups are not identical. Taken together, the results of both previous research (e.g., Shirai et al., 2012) and the present study imply that vection develops slowly and gradually throughout life. Although the present results do not explain why stronger vection occurs in younger individuals, we can speculate about the factors that may contribute to stronger vection in younger individuals.

The simplest interpretation may be that differences in sensitivity to visual motion between younger and older individuals result in difference in the perceived magnitude of vection. Indeed, many studies have shown that children are less sensitive to visual motion than are adults and that visual motion perception develops beyond childhood. For instance, even 5-year-old children are significantly less sensitive to visual motion than are adults (e.g., Ellemberg et al., 2003, 2004), and visual motion sensitivity gradually increases until at least about 14–15 years of age (e.g., Schrauf et al., 1999; Hadad et al., 2011). By the age of 15–17 years, cortical responses to visual motion patterns are largely equivalent to those of adults (Bucher et al., 2006). Because the ability to detect and perceive visual motion is one of the key prerequisites of vection, the protracted development of visual motion sensitivity may affect the long-term development of vection. However, there is no valid reason for assuming that lower motion sensitivity (and thus weaker or more ambiguous perceptions of visual motion) contributes to stronger vection. Thus, it is unlikely that the difference between children's and adults' visual motion sensitivity accounts for the observed difference in vection between children and adults.

A more plausible interpretation of our results concerns children's immature ability to use non-visual information to perceive or control self-movement. Non-visual information, such as vestibular and/or proprioceptive sensations, can also be effective cues for locomotor actions or postural control. Generally, however, previous research has shown that young children tend to rely on visual rather than on non-visual information to maintain stability in locomotion or postural control, and it takes a long time (although the precise period remains unclear, it is more than 8 years from birth) to acquire adult-level ability to use non-visual information for such motor actions (see the review by Assaiante, 1998). Indeed, previous studies have reported that vestibular and proprioceptive sensations are less effective for maintaining postural control in even children around elementary school and junior high school age than in adults (e.g., Hirabayashi and Iwasaki, 1995; Rival et al., 2005). These previous findings imply that younger children rely more on visual than on non-visual information for the perception of self-motion. Thus, the children who have participated in this and other studies on vection may have perceived stronger self-motion than did the adults because the experimental condition relied solely on a pattern of visual motion.

Alternatively, the stronger vection in younger individuals may be common to human beings in many age groups. For instance, Haibach et al. (2009) found that stronger vection occurred in younger (mean age, 18.5 years) than in older (mean ages, 64.9 and 75.0 years) adults. Research investigating the development of vection in high school- or middle-aged individuals should examine this possibility. Additionally, direct comparisons of the experiences of vection among people of different ages (i.e., from toddlerhood to old age) should provide a more comprehensive understanding of the development of vection.

Another possible but more negative interpretation of the present results is that the children (but not the adults) tended to report greater magnitude of vection than they actually experienced such that the obtained results may not reflect the “real” difference in the magnitude of vection between children and adults. We instructed participants that their actual daily bodily movement should correspond to the rating representing the greatest magnitude (the right edge of the scale) and that the state of their own body at rest should correspond to the weakest magnitude (the left edge of the scale). Thus, in theory, the participants could refer to their daily experience with their own bodily movements as the frame of reference for estimating the magnitude of vection. Indeed, the present results replicated a well-known feature of vection, namely that a smaller visual stimulus reduces the magnitude of vection (Brandt et al., 1973; Seno et al., 2013a,b) in both children and adults. This means that the experimental procedure used in the present study appears to have been appropriate and successful. Nonetheless, the possibility of inaccurate reporting discussed herein is critical and is difficult to resolve. Indeed, even if observers were provided with more concrete references for their magnitude estimations before the experimental sessions, we still could not confirm whether these references were used appropriately to estimate subjective experiences. The possible negative interpretation raised here is an important consideration for discussions of vection studies that rely on participants' subjective reports about their experiences of vection, and it should be addressed in future empirical studies.

It should be noted that the magnitude of vection reported in the present study is higher than that found in a previous study (Shirai et al., 2012) that used a similar experimental procedure. Indeed the mean magnitudes were 89.8 (*SD* = 5.2) for junior high school students and 75.4 (*SD* = 11.7) for adults in the present study, whereas they were 74.3 (*SD* = 21.9) for elementary school students and 55.2 (*SD* = 13.5) for adults in the previous study. This discrepancy between the present and previous results may derive from differences in the experimental designs of the two studies. Whereas the participants in the present study engaged in both the large-field and small-field conditions, the participants in the study conducted by Shirai et al. (2012) engaged in only one experimental condition, which corresponded to the large-field condition used in the present study. The participants in the present study could use their experience under one condition as a reference to estimate the magnitude of vection under the other condition. For instance, a participant who experienced vection under both the small-field (weaker vection) and large-field (stronger vection) conditions may have compared these experiences when reporting the magnitude of the vection in each trial. As a result, the difference between experiences of weaker and stronger vection may have been enhanced (stronger vection tended to be reported as being of a higher magnitude and weaker vection tended to be reported as being of a smaller magnitude) in the present study.

Finally, it is important to note the observed dissociation among the three measures, i.e., latency, duration, and magnitude. As noted in the Introduction and the Materials and Methods sections, we obtained three different vection measures (latency, duration, and magnitude), which, consistent with a substantial amount of previous research, independently reflected three different aspects of vection. For example, Seno et al. (2013a) presented vection stimuli to observers who were wearing iron or wooden clogs. The effect of such a burden (iron clogs) was strongly evident in reports of magnitude but weakly evident in measures of latency and duration. Reports of magnitude may be more sensitive indicators of differences in vection than are the other two measures. This possibility should be further examined in the future. In any event, the three measures reflected three different aspects of vection. It is our opinion that even though the effect was evident in only one measure, this may be sufficient proof of the modulation of vection.

## Concluding remarks

Junior high school students reported stronger vection than did adults. This finding is related to the fact that these students are at a midpoint in their development. This study revealed a connection between research on development and that on vection, and we hope that this article will serve as a foundation for future vection research.

## Author contributions

Nobu Shirai, Tomoko Imura, and Takeharu Seno designed the experiment; Rio Tamura collected data; Nobu Shirai and Rio Tamura analyzed data; Nobu Shirai, Tomoko Imura, Rio Tamura, and Takeharu Seno discussed the results; Nobu Shirai, Tomoko Imura, and Takeharu Seno wrote the manuscript.

### Conflict of interest statement

The authors declare that the research was conducted in the absence of any commercial or financial relationships that could be construed as a potential conflict of interest.
